# The Effect of Germination on Antinutritional Components, In Vitro Starch and Protein Digestibility, Content, and Bioaccessibility of Phenolics and Antioxidants of Some Pulses

**DOI:** 10.1002/fsn3.70103

**Published:** 2025-05-08

**Authors:** Neşe Yılmaz Tuncel, Havva Polat Kaya, Fatma Betül Sakarya, Ali Emre Andaç, Fatma Korkmaz, Gulay Ozkan, Necati Barış Tuncel, Esra Capanoglu

**Affiliations:** ^1^ Faculty of Applied Sciences, Department of Food Technology Çanakkale Onsekiz Mart University Çanakkale Türkiye; ^2^ Faculty of Chemical and Metallurgical Engineering, Department of Food Engineering Istanbul Technical University Istanbul Türkiye; ^3^ Faculty of Engineering, Department of Food Engineering Çanakkale Onsekiz Mart University Çanakkale Türkiye; ^4^ Faculty of Engineering, Department of Food Engineering Balıkesir University Balıkesir Türkiye

**Keywords:** legumes, phytic acid, saponin, sprouting, tannin, trypsin inhibitors

## Abstract

The objective of this study was to investigate the impact of 24‐ and 48‐h germination on antinutrient levels (phytic acid, trypsin inhibitors, saponins, and tannins), in vitro starch and protein digestibility, and the content and bioaccessibility of phenolic compounds and antioxidants in chickpeas, peas, mung beans, and lentils. Germination resulted in reductions of phytic acid by up to 75.65% and trypsin inhibitor activity by up to 39.20% in the pulses studied. In contrast, saponin levels showed a significant increase, rising nearly threefold with germination, while mung beans exhibited an exceptional 27‐fold increase. Tannins decreased in lentils (2.6‐fold) and mung beans (5.8‐fold), increased in peas (1.6‐fold), and remained unchanged in chickpeas following germination. In vitro protein digestibility generally increased with germination, reaching up to 4.40%, except in peas, where a decline was observed. Germination significantly enhanced total digestible starch content while reducing resistant starch in all pulses except chickpeas. Mung beans exhibited the highest total phenolic content and antioxidant capacity, followed by lentils. Although germination significantly elevated total phenolic content in all pulses, this increase did not always align with antioxidant capacity outcomes. Additionally, germination led to a decline in the bioaccessibility of phenolics. However, the amount of phenolic compounds progressively increased during gastric and intestinal digestion, with intestinal digestion further enhancing the total antioxidant capacity of the pulses.

## Graphical abstract

Germination reduced phytic acid (7.03%–75.65%) and trypsin inhibitor activity (4.72%–39.20%), while its effects on tannins and saponins varied. It generally improved in vitro protein digestibility except in peas and increased TDS while decreasing resistant starch, except in chickpeas. Although germination increased total phenolic content, it lowered its bioaccessibility in all pulses.

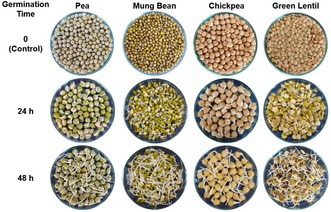

**Abbreviations**
BAEE: N‐Benzoyl‐L‐Arginine Ethyl Ester
BAPNA: *N*_α_‐Benzoyl‐DL‐arginine *p*‐nitroanilide
CUPRAC: Copper (II) ion reducing antioxidant capacity
DMACA: 4‐(dimethylamino)cinnamaldehyde
GAE: Gallic acid equivalents
GD: Gastric digestion
ID: Intestinal digestion
IVPD: In vitro protein digestibility
RDS: Rapidly digestible starch
RS: Resistant starch
SDS: Slowly digestible starch
TAC: Total antioxidant capacity
TDS: Total digestible starch
TE: Trolox equivalents
TIA: Trypsin inhibitor activity
TIU: Trypsin inhibitor units
TP: Total phenolics
UD: Undigested

## 1 Introduction

Pulses, which belong to the *Leguminosae* family, rank as the second‐most consumed crop by humans after cereals (Samtiya et al. 2020). They are an affordable source of protein, crucial for meeting the dietary needs of large populations, particularly in developing countries. Renowned for their high protein, fiber, phenolic content, and antioxidants, pulses play a vital role in providing a balanced and nutritious diet (Liu et al. 2020). The antioxidant properties of phenolic compounds in pulses have drawn considerable attention due to their potential in preventing chronic diseases (Xu et al. 2018). Additionally, pulses offer functional properties such as thickening, gelling, and water‐ and oil‐holding when incorporated into food formulations as ingredients (Sofi et al. 2023). In recognition of their health benefits, the Food and Agriculture Organization (FAO) of the United Nations designated 2016 as the International Year of Pulses under the theme Nutritious Seeds for a Sustainable Future (FAO 2016). In addition to their nutritional benefits, pulses also play a crucial role in soil fertility through nitrogen fixation, aiding in soil maintenance and restoration.

Despite the recognized nutritional benefits of pulses, their integration into diets may be impacted by cooking challenges, the presence of antinutritional compounds, low protein and starch digestibility, poor mineral bioavailability, and strong flavors (Ghavidel and Prakash 2007). To address these issues, the food industry has implemented various treatments, including germination, boiling, fermentation, extrusion, roasting, infrared and microwave heating, and enzymatic modification on whole pulses and their derivatives (Patterson et al. 2017; Sofi et al. 2023). Among these, germination stands out as a green, sustainable, and effective processing technique that improves nutritional value, raises phenolic compound levels, enhances the functional properties of pulses for diverse food uses, reduces cooking time, and lowers antinutritional factors (Chinma et al. 2022; Romano et al. 2024; Setia et al. 2019; Sofi et al. 2023; Xu et al. 2018). Germination processes are generally categorized into short‐term (24–72 h) and long‐term (72–168 h), with long‐term germination facing challenges such as microbial safety and reduced yield of raw materials (Chinma et al. 2022; Setia et al. 2019). Therefore, short‐term germination (24–72 h) is more commonly applied. Despite its potential, the specific effects of short‐term germination on antinutritional compounds and the digestibility and bioaccessibility of nutrients are still not completely clear. This study aimed to examine the changes in antinutrients such as phytate, trypsin inhibitors, saponins, and tannins, along with the in vitro digestibility of starch and protein, as well as the content and bioaccessibility of phenolics and antioxidants during germination in a simulated digestion system.

## 2 Materials and Methods

### 2.1 Materials

Dry seeds of chickpea ( *Cicer arietinum* L., Koçbaşı variety), lentil ( *Lens culinaris* L., green lentil cultivar), pea ( *Pisum sativum* L., green pea cultivar), and mung bean ( *Vigna radiata* L., green mung bean cultivar) were obtained from supermarkets.

### 2.2 Chemicals

All chemicals used in the analytical procedures were of analytical grade and purchased from Merck (Darmstadt, Germany) or Sigma‐Aldrich (St. Louis, MO, USA).

### 2.3 Germination Procedure

**FIGURE 1:**
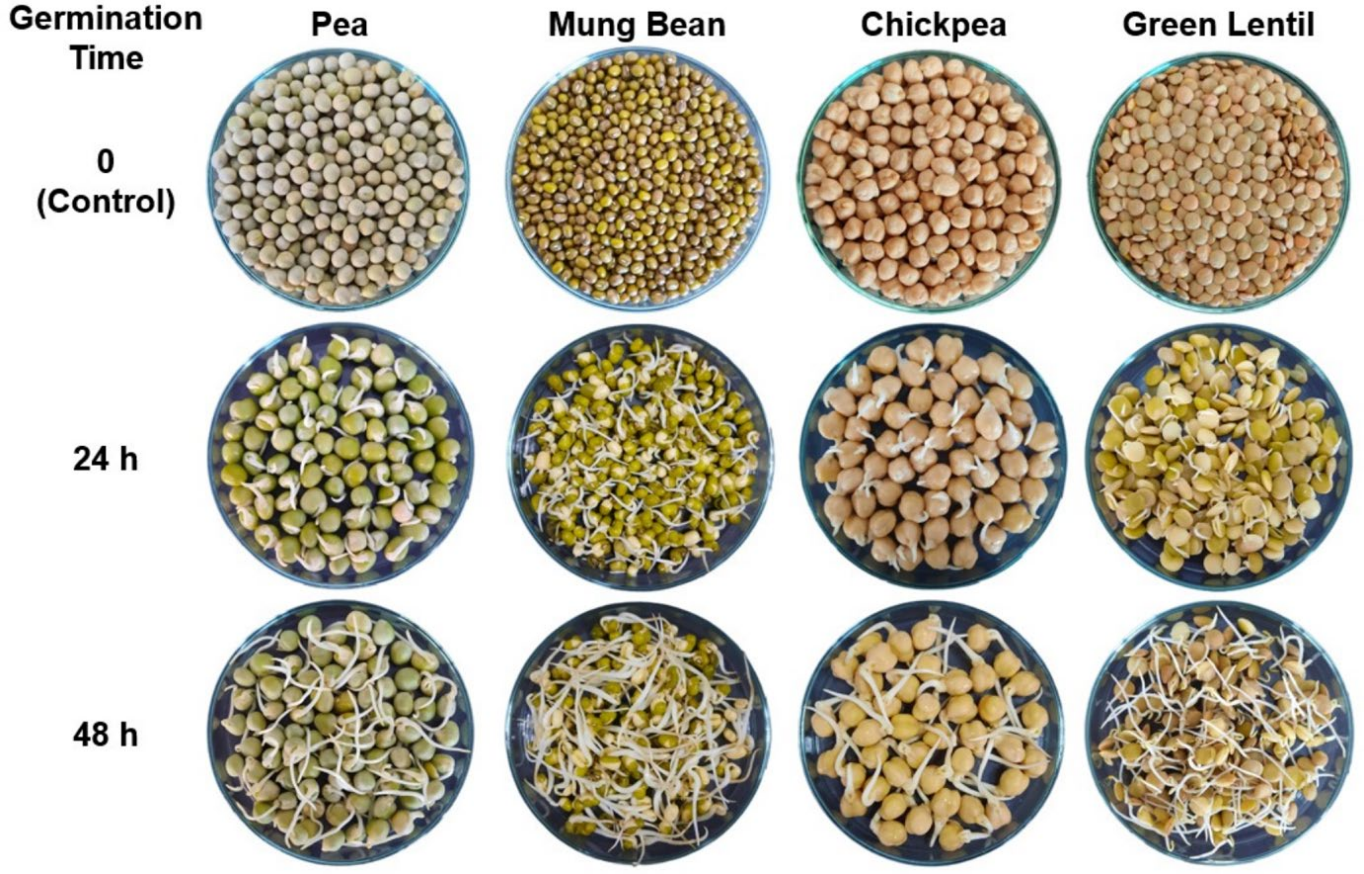
Photographs of the raw and germinated pulses.

The germination process for the pulses was carried out following the method outlined by Xu et al. (2018). The pulse seeds were soaked in a 1% NaOCl solution for 30 min and subsequently rinsed with tap water until a neutral pH was reached. Afterward, the seeds were hydrated in darkness at 25°C for 24 h and then incubated in a climate‐controlled cabinet (Nüve, TK 120; Ankara, Turkey) at 25°C in the dark for either 24 or 48 h (Figure 1). After germination, the seeds were dried in a laboratory dryer (Tre Spade Atacama, F77000; Torino, Italy) at 50°C until their moisture content dropped below 10%. Both control (ungerminated) and germinated seeds were ground for 3.5 min using a laboratory mill (Retsch, GM 200; Haan, Germany) and then passed through a 500 μm sieve. All samples were stored in the dark at −18°C for later analysis.

### 2.4 Determination of Antinutritional Compounds

#### 2.4.1 Phytic Acid

The phytic acid content of the pulse flours was determined using the standard AOAC method (method no: 986.11) (AOAC 2003). In brief, an extract prepared using diluted HCl (2.4%) was combined with EDTA‐NaOH and then loaded onto an ion‐exchange column. Phytate was digested using a mixture of sulfuric (95.0%–98.0%) and nitric acids (65%), followed by elution with a 0.7 M NaCl solution. After the reaction, the residue was treated with a molybdate solution and sulfonic acid reagent and then incubated at 25°C for 15 min. Absorbance values were measured at 640 nm using a UV–VIS spectrophotometer (Shimadzu, UV‐160A; Kyoto, Japan).

#### 2.4.2 Trypsin Inhibitors

The trypsin inhibitory activity (TIA) of the pulse flours was assessed following the half‐volume (5 mL) method (Liu 2019). Once trypsin inhibitors were extracted from the pulse flours using 10 mM NaOH, the extract was appropriately diluted to achieve trypsin inhibition ranging from 30% to 70%. The diluted suspensions were then combined with *N*_α_‐Benzoyl‐DL‐arginine *p*‐nitroanilide (BAPNA) (Sigma code: B4875) as the substrate and incubated at 37°C for 10 min in the presence of the trypsin enzyme (salt‐free, porcine pancreas ≥ 13,000–20,000 BAAE units/g protein‐ One BAEE [N‐Benzoyl‐L‐Arginine Ethyl Ester (BAEE) Hydrolysis Units] unit will produce a change in delta A253 of 0.001 per min with BAEE as substrate at pH 7.6 at 25°C). After exactly 10 min, the reaction was stopped by adding 1 mL of a 30% acetic acid solution. The absorbance was measured at 410 nm using a UV–VIS spectrophotometer, and the TIA was expressed in trypsin inhibitor units (TIU) per milligram of the sample.

#### 2.4.3 Saponin

The method specified by Antoine et al. (2022) was applied to determine the saponin content in the samples. Saponins were extracted from 0.5 g of pulse flours using 5 mL of an 80% methanol solution. The sample was mixed on an incubated shaker (Jeio Tech, IS‐971‐R; Seoul, Korea) at 25°C for 24 h, then centrifuged (Nüve, NF 800R; Ankara, Turkey) at 2000× *g* for 5 min at 4°C. The saponin extract (0.2 mL) was combined with 80% methanol (0.3 mL), 8% vanillin (0.5 mL), and 72% sulfuric acid (5 mL) solutions, then incubated at 60°C for 10 min. The absorbance values were recorded at 544 nm, employing an 80% methanol solution as the reference. The results were reported in terms of soyasaponin I equivalents.

#### 2.4.4 Tannin

Tannins were analyzed using a spectrophotometric method described by Antoine et al. (2022). Two hundred milligrams of pulse flour were extracted with 20 mL of distilled water and centrifuged at 4200× *g* and 4°C for 4 min. Then, 4 mL of tannin extract was mixed with 2 mL of methanol solution (50:50, v/v) and 1 mL of DMACA (4‐(dimethylamino)cinnamaldehyde) solution and kept in the dark for 30 min. Absorbance was measured at 640 nm using a methanol solution (50:50, v/v) as the reference. The results were expressed in terms of catechin equivalents.

### 2.5 In Vitro Protein Digestibility

The in vitro protein digestibility (IVPD) of the pulses was measured using the method described by Guldiken et al. (2022). The pulse flour dispersion (6.25 mg protein/mL) was adjusted to pH 8.0 using 0.1 M NaOH and then placed in a water bath (Memmert, WNB 22‐SV 1422; Schwabach, Germany) at 37°C for 1 h. After adding 1 mL of the multi‐enzyme solution consisting of [trypsin (Sigma code: T0303) (1.6 mg/mL), chymotrypsin (Sigma code: C4129) (3.1 mg/mL), and protease (Sigma code: P5147) (1.3 mg/mL) in 10 mL water, pH 8.0] to the sample dispersion, the pH change was monitored for 10 min $\left({\mathrm{\Delta pH}}_{\left(10\;\min\right)}\right)$. The following equation was used to calculate IVPD

(1) $$\text{IVPD}\;\left(\%\right)=65.66+18.10\times {\mathrm{\Delta pH}}_{\left(10\;\min\right)}$$

### 2.6 In Vitro Starch Digestibility

The in vitro starch digestibility of the pulses was assessed using the method described by Englyst et al. (1992) with a digestible and resistant starch assay kit (K‐DSTRS Kit; Megazyme, International, Bray, Ireland). Briefly, the amount of glucose released at specific time points during controlled enzymatic hydrolysis was determined using a spectrophotometer at 510 nm. The percentages of rapidly digestible starch (RDS), slowly digestible starch (SDS), and total digestible starch (TDS) were measured after incubation with α‐amylase and amyloglucosidase enzymes in a water bath at 37°C for 20, 120, and 240 min, respectively. Moreover, resistant starch (RS) was calculated as the starch remaining unhydrolyzed after 240 min of incubation.

### 2.7 Extraction of Total Phenolics

The total phenolics (TP) of the samples were extracted according to the previous study of Capanoglu et al. (2008). Two grams of ground samples were weighed into 15 mL test tubes, and 5 mL of methanol solution (75:25, v/v) was added. The mixture was vortexed for 10 s and then incubated at 25°C for 15 min in an ultrasonic bath (USC900TH; VWR, Radnor, PA, USA). At the end of this period, the prepared mixtures were centrifuged for 10 min at 4°C at 2000× *g* (Universal32R; HettichZentrifugen, Tuttlingen, Germany). This extraction procedure was repeated with the collected supernatants. The volumes of the two collected upper phases were combined and adjusted to 10 mL. The samples were then stored at −20°C for further analysis.

### 2.8 In Vitro Gastrointestinal Digestion Simulation

The protocol reported by Minekus et al. (2014) was conducted with minor modifications to simulate the in vitro gastrointestinal digestion system. Buccal, gastric, and intestinal digestion juices were prepared based on the aforementioned protocol. For simulating oral digestion, 5 g of sample, composed of 1 g of pulse flour and 4 g of distilled water, was combined with 4 mL of salivary juice, 25 μL of 0.3 mol/L CaCl_2_, and 975 μL of distilled water. This mixture was incubated in a water bath shaker (Memmert SV1422; Memmert GmbH & Co. Nürnberg, Germany) at 37°C for 2 min.

To simulate gastric digestion, the remaining oral bolus was mixed with 7.5 mL of gastric juice, 1.6 mL of pepsin solution (417 μkat/mL), and 5 μL of 0.3 mol/L CaCl_2_, and the pH of the prepared samples was adjusted to 3.0 with 0.2 mL of 1 mol/L HCl. Finally, the volume of the mixture was adjusted to 20 mL with distilled water and kept in a water bath shaker for 2 h at 37°C. At the end of the 2 h incubation period, 5 mL of aliquots from each sample were separated to analyze gastric digestion.

Then, 8.25 mL of intestinal juice, 3.75 mL of pancreatin (13 μkat/mL), 1.875 mL of 160 mmol/L bile, and 30 μL of 0.3 mol/L CaCl_2_ were added to the remaining gastric chyme. The pH of the mixture was adjusted to 7.0 with 1 mol/L NaOH. The final volume was completed to 30 mL and kept in a water bath shaker for 2 h at 37°C. Following the in vitro gastrointestinal digestion, all samples were cooled by submerging them in an ice bath. The residual and bioaccessible fractions were then separated by centrifugation (Megafuge 8R; Thermo Scientific, Darmstadt, Germany) at 11,500× *g* for 30 min at 4°C. The supernatants were stored at −20°C until they were subjected to spectrophotometric analysis.

To eliminate any interference caused by the enzymes and buffers utilized in the in vitro digestion protocol, a blank sample (the same amount of distilled water substituted for samples) was also used to simulate gastrointestinal digestion.

### 2.9 Phenolics and Antioxidants

#### 2.9.1 Total Phenolics

TP content of all samples was determined according to the Folin‐Ciocalteau method (Singleton and Rossi 1965). Absorbance values of the mixtures (15 μL of sample, 112.5 μL of 0.2 N Folin‐Ciocalteau reagent, and 112.5 μL of 6% sodium carbonate (w/v)) were measured after 60 min of incubation at 25°C in the dark by using a spectrophotometer (BioTek Instruments; Synergy HT) at 765 nm. A calibration curve was used (*R* ^2^ = 0.9979, *y* = 9.6217*x* − 0.033) to determine the TP content of the samples, and the results were expressed as mg gallic acid equivalents (GAE)/100 g.

#### 2.9.2 Antioxidant Capacity

The total antioxidant capacity (TAC) of the samples was determined by the copper(II) ion reducing antioxidant capacity (CUPRAC) method of Apak et al. (2014). In brief, 7 μL of extract was mixed with 70 μL of 10 mM copper(II) chloride, 70 μL of 7.5 mM neocuproine, 70 μL of 1 mM ammonium acetate (pH 7.0), and 70 μL of distilled water, respectively. The mixture was shaken for 10 s at room temperature and then incubated in the dark at 25°C for 30 min. After incubation, absorbance was measured at 450 nm. A calibration curve (*R* ^2^ = 0.991, *y* = 1.1684*x* + 0.0038) was used to determine the TAC of the samples, with results expressed as mg Trolox equivalents (TE)/100 g.

### 2.10 Statistical Analysis

One‐way ANOVA analysis was performed using Minitab (ver. 20; StatSoft Inc., Tulsa, OK, USA) to evaluate the effects of germination. Tukey's test was used for multiple comparisons, with a significant difference defined at *p* < 0.05. All measurements were performed in at least three replicates, and the results were reported as mean ± standard error.

## 3 Results and Discussion

### 3.1 Antinutritional Factors

#### 3.1.1 Phytic Acid

Phytic acid serves as the primary source of phosphorus in seeds and is considered one of the main anti‐nutritional factors that inhibit optimal nutrient absorption (Samtiya et al. 2020). It exhibits a marked ability to bind minerals, especially calcium, and is associated with the inhibition of digestive enzymes (proteases and α‐amylases) (Erba et al. 2019). The phytic acid content in ungerminated chickpeas, lentils, and peas was similar, ranging between 368 and 382 mg/100 g. However, mung beans had the lowest phytic acid content among all control pulses, with 174.70 mg/100 g (Table 1). Varying levels of phytic acid have been reported in the literature for pulses, 404–1400 mg/100 g for chickpeas (Bueckert et al. 2011; Shi et al. 2018), 420–770 mg/100 g for peas (Chigwedere et al. 2023), 612–1191 mg/100 g for lentils (Sinkovič et al. 2022), and 441–990 mg/100 g for mung beans (Dahiya et al. 2015; Tajoddin et al. 2011). In general, the phytic acid levels reported in the literature for mung beans and peas are lower compared to those of other pulses. Variations in phytic acid content can be influenced by factors such as variety or cultivar, climate, geographical location, irrigation practices, soil characteristics, cropping season, and analytical methods used (Shi et al. 2018). Germination resulted in a 7.03%–10.66% decrease in the phytic acid content of chickpeas, although this change was statistically insignificant (*p* > 0.05). Similarly, Erba et al. (2019) observed a small decrease (approximately 5%) in the phytic acid content of chickpeas after germination at 22°C for 3 days. However, it was observed that germination caused a significant decrease in the phytic acid content of lentils, peas, and mung beans (*p* < 0.05). Germination for 24 and 48 h resulted in the largest decrease in phytic acid content in lentils, with reductions of 55.87% and 75.65%, respectively. Peas experienced a decrease of 21.06%–44.12%, and mung beans showed a reduction of 30.39%–39.74% after 24 and 48 h of germination, respectively. The duration of germination was positively associated with phytate breakdown, showing a pronounced effect in lentils. Many researchers have also reported notable decreases in the phytic acid content of pulses due to germination (Erba et al. 2019; Liberal et al. 2024; Pal et al. 2017).

**TABLE 1:** Antinutritional compounds in raw and germinated pulses*.

Pulse type	Germination time (h)	Phytic acid (mg/100 g)	Trypsin inhibitors (TIU/mg)	Saponin (mg/100 g)	Tannin (mg/100 g)
Chickpeas	Control	382.80 ± 17.60^a^	6.26 ± 0.05^a^	21.37 ± 1.75^b^	49.45 ± 3.77^a^
	24	355.90 ± 34.30^a^	6.00 ± 0.05^b^	20.74 ± 1.34^b^	51.50 ± 3.79^a^
	48	342.00 ± 42.30^a^	5.52 ± 0.03^c^	29.34 ± 1.20^a^	51.85 ± 5.41^a^
Lentils	Control	375.50 ± 15.10^a^	4.59 ± 0.07^a^	16.87 ± 1.00^b^	25.82 ± 0.82^a^
	24	165.70 ± 24.10^b^	4.16 ± 0.02^b^	20.14 ± 2.09^b^	13.17 ± 0.52^b^
	48	91.42 ± 1.88^c^	3.14 ± 0.05^c^	33.52 ± 2.85^a^	9.88 ± 2.58^b^
Peas	Control	368.11 ± 3.37^a^	1.27 ± 0.12^a^	12.20 ± 1.78^b^	4.57 ± 0.48^b^
	24	290.57 ± 5.81^b^	1.25 ± 0.11^a^	18.05 ± 1.73^b^	3.89 ± 0.53^b^
	48	205.70 ± 11.40^c^	1.21 ± 0.02^a^	33.48 ± 2.14^a^	7.34 ± 0.29^a^
Mung beans	Control	174.70 ± 13.20^a^	5.97 ± 0.01^a^	2.28 ± 0.71^c^	16.97 ± 1.45^a^
	24	121.61 ± 0.81^b^	5.31 ± 0.24^b^	19.25 ± 0.61^b^	8.08 ± 4.19^ab^
	48	105.29 ± 5.23^b^	3.63 ± 0.06^c^	61.20 ± 0.23^a^	2.89 ± 0.26^b^

* Means followed by different small letters (a, b, c) within each column are significantly different (*p* < 0.05).

The reduction in phytic acid content due to germination is mainly attributed to increased phytase activity as well as the leaching of water‐soluble phytate during soaking (Elliott et al. 2022; Sofi et al. 2023). The degradation products of phytic acids, such as inositol monophosphates or diphosphates, exhibit reduced or negligible ability to chelate minerals and form insoluble complexes. The degree of phytate reduction can vary depending on the species, cultivar, germination conditions, and native phytase activity (Elliott et al. 2022). The lower phytate reduction observed in chickpeas compared to other pulses germinated under the same conditions may be linked to their lower endogenous phytase activity.

#### 3.1.2 Trypsin Inhibitors

Trypsin inhibitors are prevalent in diverse organisms, but they are especially concentrated in pulses. Trypsin inhibitors are identified as antinutrients due to their ability to bind to proteases, which disrupts the enzymes' function in breaking down proteins and, therefore, affects the absorption and digestion of amino acids (Liberal et al. 2024). TIA in ungerminated control samples was 6.26 TIU/mg in chickpeas, 5.97 TIU/mg in mung beans, 4.59 TIU/mg in lentils, and 1.27 TIU/mg in peas (Table 1). While the variability in methods and units used complicates direct comparisons with TIA data from the literature, similar findings have been reported (Shi et al. 2017).

The highest and the lowest TIA were observed in chickpeas and peas, respectively, in both control and germinated samples. Germination led to a significant decrease in TIA for chickpeas, lentils, and mung beans (*p* < 0.05), while its effect on TIA in peas was not statistically significant (*p* > 0.05). Mung beans showed the greatest reduction in TIA among the pulses tested, decreasing by 11.06% and 39.20% after 24 and 48 h of germination, respectively. The reduction in TIA was 31.60% for lentils and 11.82% for chickpeas after 48 h of germination. However, TIA in peas decreased only from 1.27 to 1.21 TIU/mg over the same period. Higher reduction rates in TIA due to germination have also been reported in the literature. Marengo et al. (2017) detected a 45% decrease in TIA of chickpeas after germination at 18°C–4°C for 3 days. Pal et al. (2017) germinated five varieties of lentils for 48 h and observed a decrease in TIA ranging from 61.86% to 71.29%.

The decrease in trypsin inhibitor activity during germination can be attributed to several factors, including enzymatic breakdown facilitated by proteolytic activity during the germination process (Elobuike et al. 2021). The germination process triggers metabolic transformations that reshape the seed's biochemical environment, enhancing nutrient availability. At the same time, changes in pH and enzyme activity contribute to the breakdown of inhibitors, while modifications in disulfide bonds promote the denaturation of trypsin inhibitors by bypassing intricate physiological pathways (Gunathunga et al. 2024).

#### 3.1.3 Saponins

Saponins, surface‐active secondary compounds predominantly found in plants, can inhibit a variety of digestive enzymes, including amylase, glucosidase, trypsin, chymotrypsin, and lipase, which may contribute to digestive health concerns (Samtiya et al. 2020). Moreover, saponins are major contributors to bitterness, with a remarkably low detection threshold of 8 mg/L (Gultekin Subasi et al. 2024). Conversely, studies have indicated that saponin consumption enhances protection against cancer risk, lowers blood cholesterol levels, influences blood sugar response, and exhibits anti‐inflammatory, hypocholesterolaemia, and immunomodulatory activities (Shi et al. 2004). As shown in Table 1, the saponin content of control pulses followed the descending order of chickpeas > lentils > peas > mung beans. Generally, the saponin levels observed in the current study were lower than those documented in the literature, likely due to variations in the pulse varieties employed, the analytical methods utilized for determination, and the agricultural practices. Stone et al. (2021) reported saponin amounts of 291–341 mg/100 g for chickpeas, 171–320 mg/100 g for lentils, and 158–170 mg/100 g for peas. Additionally, Lee et al. (2011) documented a saponin content of 431 mg/100 g for mung beans.

Unlike other antinutrients, the saponin content in pulses did not decrease after germination; instead, it increased. After 24 h of germination, there was no significant change (*p* > 0.05) in saponin content observed in chickpeas, lentils, and peas, whereas a significant increase was observed in mung beans (*p* < 0.05) (Table 1). However, after 48 h of germination, there was a notable increase in saponin content across all pulses (*p* < 0.05). Of all the pulse samples, germinated mung beans exhibited a dramatic rise in saponin content, being 8 to 28 times higher compared to the control sample (Table 1). A general increasing trend in saponin content with germination has also been documented in the literature. It has been reported that the saponin content of chickpeas (Pérez‐Ramírez et al. 2023), soybeans (Miglani and Sharma 2018), and black beans (Cardador‐Martínez et al. 2020; Guajardo‐Flores et al. 2012) increases with germination. Sprouts have been found to contain higher levels of saponins compared to cotyledons or seed coats (Guajardo‐Flores et al. 2012; Singh et al. 2017). Guajardo‐Flores et al. (2012) noted that while saponin concentration in the seed coat decreases during the first day of germination, it subsequently increases as the proportion of sprouts relative to the total weight of the germinated seeds grows. Additionally, Cardador‐Martínez et al. (2020) suggested that the increase in saponin content might be attributed to the activation or synthesis of enzyme systems involved in producing secondary compounds and altering the seed structure.

#### 3.1.4 Tannins

Tannins are phenolic compounds soluble in water that hinder the breakdown of proteins during digestion by deactivating various digestive enzymes. This occurs through the formation of multiple hydrogen bonds between the hydroxyl group of tannins and the carbonyl group of proteins, leading to the creation of both reversible and irreversible complexes between tannins and proteins (Liberal et al. 2024). In the control pulses, tannin content varied from 4.57 to 49.45 mg/100 g, with peas showing the lowest and chickpeas the highest content (Table 1). The tannin contents reported in the literature for pulses vary widely, likely due to differences in the plant variety, environmental conditions, specific plant part analyzed, maturity of the material, and the extraction and analytical methods used.

In this study, no consistent trend in tannin content was observed in response to germination. The tannin content of chickpeas remained unchanged after both 24 and 48 h of germination (*p* > 0.05). This finding is consistent with Hithamani and Srinivasan (2014), who also reported no significant change in chickpea tannin content after 48 h of germination at 25°C. Conversely, germination resulted in a significant decrease in tannin content in lentils and mung beans (*p* < 0.05). Specifically, after 24 h of germination, tannin content in lentils and mung beans decreased by approximately 50%. This reduction became more pronounced after 48 h, with tannin content dropping by 61.62% for lentils and 70.51% for mung beans. Similarly, Pal et al. (2017) reported a decrease of 58.57%–66.52% in tannin content across five lentil varieties after 48 h of germination. Other researchers also observed a decreasing trend in tannin content with germination for lentils (Liberal et al. 2024) and mung beans (Elobuike et al. 2021). This decrease has been linked to increased polyphenol oxidase activity during germination (Sofi et al. 2023). James et al. (2020) suggested that the decline in tannin content might be due to interactions between tannins and proteins, carbohydrates, and enzymes within the plant matrix.

In contrast, an unexpected increase in tannin content was noted in peas after 48 h of germination (*p* < 0.05). James et al. (2020) also observed a slight rise in tannin content in pigeon peas after 2–4 days of germination.

In this study, the variation in tannin levels in the tested pulses as a result of germination is linked to their initial tannin content. Chickpeas, which had the highest initial tannin content (49.45 mg/100 g), were unaffected by the germination process (*p* > 0.05). In contrast, peas, with the lowest initial tannin content (4.57 mg/100 g), showed an increase in tannins. Lentils and mung beans, with moderate initial tannin levels (25.82 mg/100 g for lentils and 16.97 mg/100 g for mung beans), experienced a decrease in tannin content due to germination. This implies that seeds with varying initial tannin levels may undergo different changes under the same germination conditions. To validate this hypothesis, it is crucial to conduct comprehensive testing using identical plant materials with different tannin concentrations.

### 3.2 In Vitro Protein Digestibility

IVPD values for raw and germinated pulses are presented in Table 2. Control pulses exhibited IVPD values ranging from 78.05% to 84.74%. Similarly, Sofi et al. (2023) reported IVPD values of 83.8% and 84.6% for two chickpea cultivars (GNG‐469 and ‐1581, respectively). Nosworthy et al. (2017) found IVPD values of 85.02% and 87.89% for chickpeas and lentils, respectively. Additionally, Park et al. (2010) documented IVPD values ranging from 79.9% to 83.5% across eight pea cultivars.

**TABLE 2:** In vitro protein digestibility of raw and germinated pulses (%)*.

Pulse type	Germination period (h)	IVPD (%)	Variation rate (%)
Chickpeas	0	80.50 ± 0.10^c^	—
	24	81.13 ± 0.05^b^	+0.78
	48	84.03 ± 0.05^a^	+4.38
Lentils	0	78.23 ± 0.05^c^	—
	24	80.41 ± 0.05^b^	+2.78
	48	81.22 ± 0.10^a^	+3.82
Peas	0	84.74 ± 0.05^a^	—
	24	82.76 ± 0.15^b^	−2.33
	48	82.77 ± 0.36^b^	−2.32
Mung beans	0	78.05 ± 0.05^c^	—
	24	80.41 ± 0.06^b^	+3.02
	48	81.49 ± 0.05^a^	+4.40

Abbreviation: IVPD, in vitro protein digestibility.

* Means followed by different small letters are significantly different (*p <* 0.05).

Germination enhanced the IVPD of chickpeas, lentils, and mung beans, whereas the IVPD of both raw and germinated peas remained comparable, with a slight decrease observed. After 24 h of germination, the lowest increase in IVPD was observed in chickpeas (0.63%), while this rate was 2.18% and 2.36% in lentils and mung beans, respectively. However, the IVPD of chickpeas increased by 3.53% after 48 h of germination, while for lentils and mung beans, the increases were 2.99% and 3.44%, respectively. Nonetheless, extended germination periods led to significant increases in IVPD in pulses, except for peas (*p* < 0.05). Chinma et al. (2022) similarly observed IVPD values of 83.10%, 86.44%, and 88.90% in Bambara groundnut flour after 24, 48, and 72 h of germination, respectively.

An increase in IVPD resulting from germination has been documented by several studies, with the primary causes identified as partial protein hydrolysis and a decrease in antinutritional factors, especially phytic acid and tannin (Ghavidel and Prakash 2007; Sofi et al. 2023). It is noteworthy that the IVPD of peas showed a minor reduction after 24 or 48 h of germination. This could be related to insufficient activation of enzymes during germination or the stability of pea proteins, which may remain unaffected by germination, causing no significant change in IVPD. Likewise, Urbano et al. (2005) utilized a multienzyme pH drop method and reported a reduction in IVPD of germinated peas over 6 days. The researchers linked this decline to the presence of pea peptides that resist proteolytic enzyme activity.

### 3.3 In Vitro Starch Digestibility

In vitro starch digestibility values for raw and germinated pulses are presented in Table 3. Germination resulted in minor and insignificant changes in the RDS content of chickpeas and peas after 48 h (*p* > 0.05). In contrast, lentils and mung beans exhibited a notable increase in their RDS content, rising up to 1.5 and 1.9 times, respectively, over the 48‐h germination period (*p* < 0.05). Additionally, the SDS content of all tested pulses increased with prolonging the germination period except for the chickpeas (*p* < 0.05) (Table 3).

**TABLE 3:** In vitro starch digestibility of raw and germinated pulses*.

Pulse type	Germination time (h)	RDS (%)	SDS (%)	TDS (%)	RS (%)
Chickpeas	0	2.40 ± 0.14^a^	11.10 ± 0.06^a^	20.24 ± 0.59^a^	13.14 ± 0.64 ^b^
	24	1.84 ± 0.05^a^	9.00 ± 0.31^c^	16.95 ± 0.73^b^	15.74 ± 0.31^a^
	48	2.10 ± 0.29^a^	10.07 ± 0.16^b^	18.38 ± 0.02^ab^	14.04 ± 0.80^ab^
Lentils	0	2.78 ± 0.07^c^	8.71 ± 0.16^c^	14.85 ± 0.04^c^	12.45 ± 0.11^a^
	24	3.39 ± 0.10^b^	17.49 ± 0.21^b^	25.27 ± 0.25^b^	12.13 ± 0.14^a^
	48	4.28 ± 0.21^a^	20.24 ± 0.27^a^	31.82 ± 0.59^a^	7.66 ± 0.16^b^
Peas	0	4.01 ± 0.14^a^	11.38 ± 0.11^c^	18.06 ± 0.55^b^	15.17 ± 0.09^a^
	24	3.35 ± 0.02^b^	14.26 ± 0.17^b^	24.29 ± 0.70^a^	14.28 ± 0.08^a^
	48	4.47 ± 0.25^a^	16.22 ± 0.24^a^	28.05 ± 1.73^a^	10.90 ± 0.37^b^
Mung beans	0	2.40 ± 0.14^c^	7.69 ± 0.04^c^	12.28 ± 0.10^c^	18.88 ± 0.22^a^
	24	3.46 ± 0.05^b^	12.62 ± 0.33^b^	20.54 ± 0.12^b^	11.66 ± 0.4^b^
	48	4.67 ± 0.05^a^	18.45 ± 0.04^a^	26.34 ± 0.45^a^	3.11 ± 0.05^c^

Abbreviations: RDS, Rapid digestible starch; RS, Resistant starch; SDS, Slowly digestible starch; TDS, Total digestible starch.

* Means followed by different small letters within each column are significantly different (*p <* 0.05).

This study assessed the RDS, SDS, and TDS fractions following incubation with starch‐digesting enzymes at 37°C for 20, 120, and 240 min. RDS represents the starch fraction that undergoes rapid and complete digestion in the gastrointestinal tract, leading to a swift increase in postprandial plasma glucose. In contrast, SDS is digested at a slower pace in the small intestine. The TDS content, which refers to the hydrolyzed starch after 240 min, significantly increased with germination in all pulses except chickpeas (*p* < 0.05). Specifically, the TDS content of lentils and mung beans increased by more than 100%, and that of peas increased by more than 50% after 48 h of germination (Table 3). Increased starch digestibility with germination is also documented in the literature, as supported by the findings from Sofi et al. (2023) for two different chickpea cultivars, Kaur et al. (2015) for mung beans, and Romano et al. (2024) for lentils. It is proposed that the reduction of antinutritional factors during germination might boost enzymatic activity, thereby improving starch digestibility (Romano et al. 2024). Moreover, the breakdown of protein and fiber structures during germination could make starch granules more accessible to enzymes (Setia et al. 2019). The increased activity of endogenous α‐amylase during germination may also play a role in the enhanced starch digestibility (Sofi et al. 2023).

The lack of expected improvement in chickpea starch digestibility may be attributed to the higher levels of antinutritional compounds present. As shown in Table 1, chickpeas had the highest concentrations of all analyzed antinutritional compounds. Variations in in vitro starch digestibility, both among different species and within a single species, may be influenced by factors such as limited enzyme activity, the size and structure of starch granules, the degree of crystallinity, the amylose‐to‐amylopectin ratio, and chain lengths (Hoover et al. 2010).

Additionally, germination led to a significant decrease in RS content, which refers to starch remaining unhydrolyzed after 240 min for all pulses except chickpeas (*p* < 0.05). Mung beans exhibited the largest reductions in RS content during both 24 h and 48 h of germination, ranging from 7.22% to 15.55%. Lentils and peas showed reductions of up to 4.79% in RS content. Previous studies have reported similar reductions in RS content in pulses such as green lentils (Romano et al. 2024), peas (Setia et al. 2019), chickpeas (Chinma et al. 2022), and mung beans (Kaur et al. 2015) following germination.

### 3.4 Total Phenolics

The effect of simulated in vitro digestion on TP of various pulses during different germination periods is shown in Table 4. Among all undigested samples, mung beans exhibited the highest TP content both before (95 ± 3 mg GAE/100 g) and after germination (156 ± 23 mg GAE/100 g). Additionally, germination led to a time‐dependent increase in TP content across the samples. Gharachorloo et al. (2013) found that the TP content of all germinated chickpeas, lentils, and white beans extracted with different solvents was significantly higher compared to ungerminated samples (*p* < 0.05). Similarly, Ramos‐Pacheco et al. (2024) observed an increase in the TP content of white and red quinoa during 0, 24, and 48 h of germination. Various exogenous and endogenous aspects, such as hormones, light, temperature, and nutrients, affect the enzymes involved in the biosynthesis of bioactive components. During germination, the activation of endogenous enzymes in pulses leads to changes in the chemical composition of lentils, beans, and peas (López‐Amorós et al. 2006; Nemhauser and Chory 2002). Additionally, the increase in phenolic compounds may be linked to the shikimic acid pathway, which contributes to phenolic biosynthesis (Kim et al. 2016).

**TABLE 4:** Content and bioaccessibility of total phenolics in raw and germinated pulses*, **, ***.

Pulse type	Germination time (h)	UD (mg GAE/100 g)	GD (mg GAE/100 g)	ID (mg GAE/100 g)	Bioaccessibility (%)
Chickpeas	Control	37 ± 5^defC2^	60 ± 11^dB2^	200 ± 22^dA2^	536 ± 59^b1^
	24	44 ± 3^defC2^	107 ± 14^bB1^	231 ± 15^bcdA1^	530 ± 34^b1^
	48	55 ± 4^cdC1^	84 ± 11^bcB2^	234 ± 20^bcdA1^	429 ± 37^bcde2^
Lentils	Control	55 ± 3^cdC1^	87 ± 16^bcB12^	252 ± 27^bcdA1^	462 ± 50^cb1^
	24	56 ± 5^cdC1^	72 ± 6^cdB2^	249 ± 24^bcdA1^	445 ± 42^bdc1^
	48	63 ± 11^cC1^	103 ± 11^bB1^	221 ± 25^cdA1^	351 ± 39^cde2^
Mung beans	Control	95 ± 3^bB2^	88 ± 18^bcB12^	286 ± 26^abA12^	302 ± 28^e1^
	24	99 ± 17^bB2^	69 ± 13^cdC2^	330 ± 37^aA1^	332 ± 37^de1^
	48	156 ± 23^aB1^	91 ± 6^bcA1^	263 ± 28^bcA2^	168 ± 18^f2^
Peas	Control	27 ± 2^fC2^	103 ± 7^bB2^	203 ± 40^dA1^	740 ± 120^a1^
	24	33 ± 6^efC2^	103 ± 4^bB2^	232 ± 22^bcdA1^	700 ± 68^a1^
	48	48 ± 4^cdeC1^	148 ± 15^aB1^	239 ± 27^bcdA1^	499 ± 57^b2^

Abbreviations: GAE, gallic acid equivalents; GD, gastric digestion; ID, intestinal digestion; UD, undigested.

* Within each column, means followed by different small cases are significantly different (*p <* 0.05).

** Within each row, means followed by different capital letters are significantly different (*p <* 0.05).

*** Within each group of samples, means followed by different numbers are significantly different (*p <* 0.05).

After gastric digestion, the TP content of all samples except mung beans increased significantly (*p* < 0.05). This rise in phenolic compound release is likely due to the acidic environment of the gastric phase, which facilitates greater phenolic release (Bermúdez‐Soto et al. 2007). A significant increase in TP content was also observed for all samples following intestinal digestion (*p* < 0.05). Previous research found that the TP content of germinated lentils significantly increased during the intestinal phase (Miyahira et al. 2022). The TP content of the samples exhibited variability during germination when subjected to in vitro simulated digestion. The bioaccessibility of TP decreased for chickpeas, lentils, and peas during all germination times. Conversely, the TP of mung beans became more bioaccessible after 24 h of germination but then significantly decreased by the end of 48 h. The bioaccessibility of phenolic compounds may be affected by factors such as the binding of phenolic compounds within the food matrix, cell wall structure, and varying concentrations in plant tissues (Miyahira et al. 2022).

### 3.5 Antioxidants

The changes in TAC of the pulses during in vitro gastrointestinal digestion are presented in Table 5. Consistent with the TP results, ungerminated mung beans had the highest TAC value at 232 ± 21 mg TE/100 g, followed by ungerminated lentils at 222 ± 33 mg TE/100 g among the undigested samples. However, there was no correlation between the TP content and TAC values of samples during germination. Germination significantly increased the TAC of undigested chickpeas and peas (*p* < 0.05), while the TAC of lentils decreased significantly, and there was no significant change in the TAC of mung beans. Guajardo‐Flores et al. (2012) reported no significant change in TAC for black beans during germination, whereas López‐Amorós et al. (2006) observed a significant increase in TAC for peas but a decrease for lentils. Germination generally enhances antioxidant activity in pulses and cereals by increasing the release and biosynthesis of phenolic acids and flavonoids through the hydrolysis of the cell wall matrix, which includes structural proteins, lignin, cellulose, hemicellulose, and pectin (Alvarez‐Jubete et al. 2010). Nonetheless, the activation of endogenous enzymes during germination leads to varying changes in the chemical structures of lentils, beans, and peas (Rao and Deosthale 1987).

**TABLE 5:** Antioxidant capacity and bioaccessibility of raw and germinated pulses*, **, ***.

Pulse type	Germination time (h)	UD (mg TE/100 g)	GD (mg TE/100 g)	ID (mg TE/100 g)	Bioaccessibility (%)
Chickpeas	0	63 ± 9^eB3^	43 ± 5^fC2^	237 ± 20^cdeA1^	377 ± 31^a1^
	24	83 ± 9^deB2^	108 ± 11^cdB1^	271 ± 43^cdA1^	327 ± 52^ab1^
	48	117 ± 13^bdcB1^	38 ± 6^fC2^	227 ± 39^cdeA1^	193 ± 33^d2^
Lentils	0	222 ± 33^aB1^	298 ± 50^aB1^	603 ± 77^aA1^	271 ± 35^bc1^
	24	130 ± 15^bcB2^	92 ± 17^deC2^	251 ± 43^cdA2^	193 ± 33^d2^
	48	156 ± 15^bB2^	108 ± 17^cdC2^	299 ± 22^cA2^	192 ± 14^d2^
Mung beans	0	232 ± 21^aB1^	110 ± 12^cdC1^	402 ± 59^bA1^	174 ± 25^de1^
	24	205 ± 23^aB1^	68 ± 5^efC2^	256 ± 26^cdA2^	125 ± 13^e2^
	48	205 ± 38^aB1^	109 ± 7^cdC1^	415 ± 38^bA1^	203 ± 19^d1^
Peas	0	61 ± 6^eB3^	90 ± 7^deA3^	71 ± 11^fB3^	118 ± 18^e2^
	24	94 ± 17^cdeC2^	135 ± 7^bcB2^	199 ± 23^deA1^	213 ± 25^cd1^
	48	131 ± 18^bcB1^	151 ± 9^bA1^	155 ± 14^efA2^	118 ± 11^e2^

Abbreviations: GD, gastric digestion; ID, intestinal digestion; TE, Trolox equivalents; UD, undigested.

* Within each column, means followed by different small cases are significantly different (*p <* 0.05).

** Within each row, means followed by different capital letters are significantly different (*p <* 0.05).

*** Within each group of samples, means followed by different numbers are significantly different (*p <* 0.05).

Following simulated gastric digestion, the results varied; however, the TAC of most samples showed a significant decrease compared to the undigested pulses (*p* < 0.05). Germination had a noticeable effect on the TAC of gastric‐digested samples, with some showing an initial increase followed by a decrease, while others displayed the opposite trend. In contrast, intestinal digestion resulted in an increase in TAC for all samples. During the intestinal phase, the rise in antioxidant potential is attributed to the formation of new oxidation products that are more effective than their precursors. Additionally, the presence of non‐phenolic antioxidants may also contribute to this increase (Rasera et al. 2023). While the bioaccessibility of TAC decreased in chickpeas and lentils with increased germination time, the TAC of peas became more available after 24 h of germination but significantly reduced after 48 h. On the other hand, in the case of mung beans, the in vitro retention of TAC slightly decreased in the 24 h germinated sample, while the 48 h germinated sample showed significantly higher TAC bioaccessibility.

## 4 Conclusion

Germination for up to 48 h resulted in reductions in phytic acid ranging from 7.03% to 75.65%, with TIA reductions varying between 4.72% and 39.20% across the pulses studied. Saponins increased, while tannin levels fluctuated depending on their initial content during germination. Control pulses exhibited IVPD values ranging from 78.05% to 84.74%. Germination improved IVPD in chickpeas, lentils, and mung beans, while peas showed similar IVPD between raw and germinated forms, with a slight decline.

Except for chickpeas, germination significantly increased TDS content and decreased RS content in all pulses. Mung beans had the highest TP content and TAC in undigested samples, followed by lentils. After intestinal digestion, all samples showed significant increases in TP content, although the bioaccessibility of TP generally decreased with germination. Germination significantly boosted TAC in undigested chickpeas and peas (*p* < 0.05), while it caused a significant decrease in lentils and had no effect on mung beans. Intestinal digestion, however, generally increased TAC in all samples.

In conclusion, the results varied based on both the pulse species and the germination conditions. The key limitations of this study include various germination conditions, such as temperature and duration, as well as genetic differences among varieties and cultivars, all of which may impact germination outcomes. A more extensive study involving different varieties under multiple conditions would enhance the depth of analysis, which is recommended for future research.

## Author Contributions

**Neşe Yılmaz Tuncel:** conceptualization (equal), formal analysis (equal), funding acquisition (lead), writing – original draft (equal). **Havva Polat Kaya:** data curation (equal), formal analysis (equal), methodology (equal). **Fatma Betül Sakarya:** data curation (equal), formal analysis (equal). **Ali Emre Andaç:** formal analysis (equal), methodology (equal), visualization (equal). **Fatma Korkmaz:** data curation (equal), visualization (equal), writing – original draft (equal). **Gulay Ozkan:** data curation (equal), writing – review and editing (equal). **Necati Barış Tuncel:** supervision (equal), writing – review and editing (equal). **Esra Capanoglu:** supervision (equal), writing – review and editing (equal).

## Ethics Statement

The authors have nothing to report.

## Conflicts of Interest

The authors declare no conflicts of interest.

## Data Availability

The data that support the findings of this study are available on request from the corresponding author.

## References

[fsn370103-bib-0001] Alvarez‐Jubete, L. , H. Wijngaard , E. K. Arendt , and E. Gallagher . 2010. “Polyphenol Composition and In Vitro Antioxidant Activity of Amaranth, Quinoa Buckwheat and Wheat as Affected by Sprouting and Baking.” Food Chemistry 119, no. 2: 770–778. 10.1016/j.foodchem.2009.07.032.

[fsn370103-bib-0002] Antoine, T. , S. Georgé , A. Leca , et al. 2022. “Reduction of Pulse “Antinutritional” Content by Optimizing Pulse Canning Process Is Insufficient to Improve Fat‐Soluble Vitamin Bioavailability.” Food Chemistry 370: 131021. 10.1016/j.foodchem.2021.131021. 34536784

[fsn370103-bib-0003] AOAC . 2003. Official Method of Analysis. 17th ed. Association of Official Analytical Chemists.

[fsn370103-bib-0004] Apak, R. , M. Özyürek , K. Güçlü , B. Bekdeşer , and M. Bener . 2014. “The CUPRAC Methods of Antioxidant Measurement for Beverages.” In Processing and Impact on Antioxidants in Beverages, edited by V. Preedy . Academic Press. 10.1016/B978-0-12-404738-9.00024-6.

[fsn370103-bib-0005] Bermúdez‐Soto, M. J. R. , F. A. Tomás‐Barberán , and M. T. García‐Conesa . 2007. “Stability of Polyphenols in Chokeberry (*Aronia melanocarpa*) Subjected to In Vitro Gastric and Pancreatic Digestion.” Food Chemistry 102, no. 3: 865–874. 10.1016/j.foodchem.2006.06.025.

[fsn370103-bib-0006] Bueckert, R. A. , D. Thavarajah , P. Thavarajah , and J. Pritchard . 2011. “Phytic Acid and Mineral Micronutrients in Field‐Grown Chickpea (*Cicer arietinum* L.) Cultivars From Western Canada.” European Food Research and Technology 233, no. 2: 203–212. 10.1007/s00217-011-1495-8.

[fsn370103-bib-0007] Capanoglu, E. , J. Beekwilder , D. Boyacioglu , R. Hall , and R. de Vos . 2008. “Changes in Antioxidant and Metabolite Profiles During Production of Tomato Paste.” Journal of Agricultural and Food Chemistry 56, no. 3: 964–973. 10.1021/jf072990e. 18205308

[fsn370103-bib-0008] Cardador‐Martínez, A. , Y. Martínez‐Tequitlalpan , T. Gallardo‐Velazquez , et al. 2020. “Effect of Instant Controlled Pressure‐Drop on the Non‐Nutritional Compounds of Seeds and Sprouts of Common Black Bean (*Phaseolus vulgaris* L.).” Molecules 25, no. 6: 1464. 10.3390/molecules25061464. 32213962 PMC7146566

[fsn370103-bib-0009] Chigwedere, C. M. , A. Stone , D. Konieczny , et al. 2023. “Examination of the Functional Properties, Protein Quality, and Iron Bioavailability of Low‐Phytate Pea Protein Ingredients.” European Food Research and Technology 249, no. 6: 1517–1529. 10.1007/s00217-023-04232-x.

[fsn370103-bib-0010] Chinma, C. E. , J. O. Abu , O. E. Adedeji , et al. 2022. “Nutritional Composition, Bioactivity, Starch Characteristics, Thermal and Microstructural Properties of Germinated Pigeon Pea Flour.” Food Bioscience 49: 101900. 10.1016/j.fbio.2022.101900.

[fsn370103-bib-0011] Dahiya, P. K. , A. R. Linnemann , M. A. J. S. Van Boekel , N. Khetarpaul , R. B. Grewal , and M. J. R. Nout . 2015. “Mung Bean: Technological and Nutritional Potential.” Critical Reviews in Food Science and Nutrition 55, no. 5: 670–688. 10.1080/10408398.2012.671202. 24915360

[fsn370103-bib-0012] Elliott, H. , P. Woods , B. D. Green , and A. P. Nugent . 2022. “Can Sprouting Reduce Phytate and Improve the Nutritional Composition and Nutrient Bioaccessibility in Cereals and Legumes?” Nutrition Bulletin 47, no. 2: 138–156. 10.1111/nbu.12549. 36045098

[fsn370103-bib-0013] Elobuike, C. S. , M. A. Idowu , A. A. Adeola , and H. A. Bakare . 2021. “Nutritional and Functional Attributes of Mungbean (*Vigna radiata* [L] Wilczek) Flour as Affected by Sprouting Time.” Legume Science 3, no. 4: e100. 10.1002/leg3.100.

[fsn370103-bib-0014] Englyst, H. N. , S. M. Kingman , and J. H. Cummings . 1992. “Classification and Measurement of Nutritionally Important Starch Fractions.” European Journal of Clinical Nutrition 46, no. Suppl 2: S33–S50. 1330528

[fsn370103-bib-0015] Erba, D. , D. Angelino , A. Marti , et al. 2019. “Effect of Sprouting on Nutritional Quality of Pulses.” International Journal of Food Sciences and Nutrition 70, no. 1: 30–40. 10.1080/09637486.2018.1478393. 29848118

[fsn370103-bib-0016] FAO . 2016. “International Year of Pulses 2016.” https://www.fao.org/pulses‐2016/en/.

[fsn370103-bib-0017] Gharachorloo, M. , B. Ghiassi Tarzi , and M. Baharinia . 2013. “The Effect of Germination on Phenolic Compounds and Antioxidant Activity of Pulses.” Journal of the American Oil Chemists' Society 90, no. 3: 407–411. 10.1007/s11746-012-2170-3.

[fsn370103-bib-0018] Ghavidel, R. A. , and J. Prakash . 2007. “The Impact of Germination and Dehulling on Nutrients, Antinutrients, In Vitro Iron and Calcium Bioavailability and In Vitro Starch and Protein Digestibility of Some Legume Seeds.” LWT‐ Food Science and Technology 40, no. 7: 1292–1299. 10.1016/j.lwt.2006.08.002.

[fsn370103-bib-0019] Guajardo‐Flores, D. , M. García‐Patiño , D. Serna‐Guerrero , J. A. Gutiérrez‐Uribe , and S. O. Serna‐Saldívar . 2012. “Characterization and Quantification of Saponins and Flavonoids in Sprouts, Seed Coats and Cotyledons of Germinated Black Beans.” Food Chemistry 134, no. 3: 1312–1319. 10.1016/j.foodchem.2012.03.020. 25005948

[fsn370103-bib-0020] Guldiken, B. , D. Konieczny , A. Franczyk , et al. 2022. “Impacts of Infrared Heating and Tempering on the Chemical Composition, Morphological, Functional Properties of Navy Bean and Chickpea Flours.” European Food Research and Technology 248, no. 3: 767–781. 10.1007/s00217-021-03918-4.

[fsn370103-bib-0021] Gultekin Subasi, B. , B. Forghani , and M. Abdollahi . 2024. “Exploring Swedish Pea Varieties Suitable for Protein Isolation, Focusing on Antinutrients and Off‐Flavors.” Journal of Food Composition and Analysis 128: 105988. 10.1016/j.jfca.2024.105988.

[fsn370103-bib-0022] Gunathunga, C. , S. Senanayake , M. A. Jayasinghe , et al. 2024. “Germination Effects on Nutritional Quality: A Comprehensive Review of Selected Cereals and Pulses Changes.” Journal of Food Composition and Analysis 128: 106024. 10.1016/j.jfca.2024.106024.

[fsn370103-bib-0023] Hithamani, G. , and K. Srinivasan . 2014. “Bioaccessibility of Polyphenols From Wheat (*Triticum aestivum*), sorghum (*Sorghum bicolor*), green Gram (*Vigna radiata*), and Chickpea (*Cicer arietinum*) as Influenced by Domestic Food Processing.” Journal of Agricultural and Food Chemistry 62, no. 46: 11170–11179. 10.1021/jf503450u. 25340251

[fsn370103-bib-0024] Hoover, R. , T. Hughes , H. J. Chung , and Q. Liu . 2010. “Composition, Molecular Structure, Properties, and Modification of Pulse Starches: A Review.” Food Research International 43, no. 2: 399–413. 10.1016/j.foodres.2009.09.001.

[fsn370103-bib-0025] James, S. , T. U. Nwabueze , J. Ndife , G. I. Onwuka , and M. Ata'Anda Usman . 2020. “Influence of Fermentation and Germination on Some Bioactive Components of Selected Lesser Legumes Indigenous to Nigeria.” Journal of Agriculture and Food Research 2: 100086. 10.1016/j.jafr.2020.100086.

[fsn370103-bib-0026] Kaur, M. , K. S. Sandhu , R. Ahlawat , and S. Sharma . 2015. “In Vitro Starch Digestibility, Pasting and Textural Properties of Mung Bean: Effect of Different Processing Methods.” Journal of Food Science and Technology 52, no. 3: 1642–1648. 10.1007/s13197-013-1136-2. 25745235 PMC4348271

[fsn370103-bib-0027] Kim, M. Y. , G. Y. Jang , Y. Lee , et al. 2016. “Free and Bound Form Bioactive Compound Profiles in Germinated Black Soybean (*Glycine max* L.).” Food Science and Biotechnology 25, no. 6: 1551–1559. 10.1007/s10068-016-0240-2. 30263444 PMC6049237

[fsn370103-bib-0028] Lee, J. H. , J. K. Jeon , S. G. Kim , S. H. Kim , T. Chun , and J.‐Y. Imm . 2011. “Comparative Analyses of Total Phenols, Flavonoids, Saponins and Antioxidant Activity in Yellow Soy Beans and Mung Beans.” International Journal of Food Science & Technology 46, no. 12: 2513–2519. 10.1111/j.1365-2621.2011.02775.x.

[fsn370103-bib-0029] Liberal, Â. , Â. Fernandes , I. C. F. R. Ferreira , A. M. Vivar‐Quintana , and L. Barros . 2024. “Effect of Different Physical Pre‐Treatments on Physicochemical and Techno‐Functional Properties, and on the Antinutritional Factors of Lentils (*Lens culinaris* spp).” Food Chemistry 450: 139293. 10.1016/j.foodchem.2024.139293. 38631207

[fsn370103-bib-0030] Liu, K. 2019. “Soybean Trypsin Inhibitor Assay: Further Improvement of the Standard Method Approved and Reapproved by American Oil Chemists' Society and American Association of Cereal Chemists International.” Journal of the American Oil Chemists' Society 96: 635–645. 10.1002/aocs.12205.

[fsn370103-bib-0031] Liu, Y. , S. Ragaee , M. F. Marcone , and E. S. M. Abdel‐Aal . 2020. “Composition of Phenolic Acids and Antioxidant Properties of Selected Pulses Cooked With Different Heating Conditions.” Food 9, no. 7: 908. 10.3390/foods9070908. PMC7404658 32664208

[fsn370103-bib-0032] López‐Amorós, M. L. , T. Hernández , and I. Estrella . 2006. “Effect of Germination on Legume Phenolic Compounds and Their Antioxidant Activity.” Journal of Food Composition and Analysis 19, no. 4: 277–283. 10.1016/j.jfca.2004.06.012.

[fsn370103-bib-0033] Marengo, M. , A. Carpen , F. Bonomi , et al. 2017. “Macromolecular and Micronutrient Profiles of Sprouted Chickpeas to Be Used Forintegrating Cereal‐Based Food.” Cereal Chemistry 94, no. 1: 82–88. 10.1094/CCHEM-04-16-0108-FI.

[fsn370103-bib-0034] Miglani, H. , and S. Sharma . 2018. “Impact of Germination Time and Temperature Onphenolics, Bioactive Compounds and Antioxidant Activity of Different Coloured Soybean.” Proceedings of the National Academy of Sciences, India Section B: Biological Sciences 88, no. 1: 175–184. 10.1007/s40011-016-0744-9.

[fsn370103-bib-0035] Minekus, M. , M. Alminger , P. Alvito , et al. 2014. “A Standardised Static In Vitro Digestion Method Suitable for Food—An International Consensus.” Food & Function 5, no. 6: 1113–1124. 10.1039/C3FO60702J. 24803111

[fsn370103-bib-0036] Miyahira, R. F. , F. de Lima Pena , G. A. Fabiano , et al. 2022. “Changes in Phenolic Compound and Antioxidant Activity of Germinated Broccoli, Wheat, and Lentils During Simulated Gastrointestinal Digestion.” Plant Foods for Human Nutrition 77, no. 2: 233–240. 10.1007/s11130-022-00970-7. 35553352

[fsn370103-bib-0037] Nemhauser, J. , and J. Chory . 2002. Photomorphogenesis. Vol. 1. Arabidopsis Book/American Society of Plant Biologists. 10.1199/tab.0054. PMC3243328 22303211

[fsn370103-bib-0038] Nosworthy, M. G. , J. Neufeld , P. Frohlich , G. Young , L. Malcolmson , and J. D. House . 2017. “Determination of the Protein Quality of Cooked Canadian Pulses.” Food Science & Nutrition 5, no. 4: 896–903. 10.1002/fsn3.473. 28748078 PMC5521049

[fsn370103-bib-0039] Pal, R. S. , A. Bhartiya , P. Yadav , et al. 2017. “Effect of Dehulling, Germination and Cooking on Nutrients, Anti‐Nutrients, Fatty Acid Composition and Antioxidant Properties in Lentil (*Lens culinaris*).” Journal of Food Science and Technology 54, no. 4: 909–920. 10.1007/s13197-016-2351-4. 28303042 PMC5336446

[fsn370103-bib-0040] Park, S. J. , T. W. Kim , and B.‐K. Baik . 2010. “Relationship Between Proportion and Composition of Albumins, and In Vitro Protein Digestibility of Raw and Cooked Pea Seeds (*Pisum sativum* L.).” Journal of the Science of Food and Agriculture 90, no. 10: 1719–1725. 10.1002/jsfa.4007. 20564440

[fsn370103-bib-0041] Patterson, C. A. , J. Curran , and T. Der . 2017. “Effect of Processing on Antinutrient Compounds in Pulses.” Cereal Chemistry 94, no. 1: 2–10. 10.1094/CCHEM-05-16-0144-FI.

[fsn370103-bib-0042] Pérez‐Ramírez, I. F. , D. E. Escobedo‐Alvarez , M. Mendoza‐Sánchez , et al. 2023. “Phytochemical Profile and Composition of Chickpea (*Cicer arietinum* L.): Varietal Differences and Effect of Germination Under Elicited Conditions.” Plants 12, no. 17: 3093. 10.3390/plants12173093. 37687340 PMC10489618

[fsn370103-bib-0043] Ramos‐Pacheco, B. S. , D. Choque‐Quispe , C. A. Ligarda‐Samanez , et al. 2024. “Effect of Germination on the Physicochemical Properties, Functional Groups, Content of Bioactive Compounds, and Antioxidant Capacity of Different Varieties of Quinoa (*Chenopodium quinoa* Willd.) Grown in the High Andean Zone of Peru.” Food 13, no. 3: 417. 10.3390/foods13030417. PMC10855556 38338552

[fsn370103-bib-0044] Rao, P. U. , and Y. G. Deosthale . 1987. “Polyphenoloxidase Activity in Germinated Legume Seeds.” Journal of Food Science 52, no. 6: 1549–1551. 10.1111/j.1365-2621.1987.tb05877.x.

[fsn370103-bib-0045] Rasera, G. B. , A. C. de Camargo , and R. J. S. Castro . 2023. “Bioaccessibility of Phenolic Compounds Using the Standardized INFOGEST Protocol: A Narrative Review.” Comprehensive Reviews in Food Science and Food Safety 22, no. 1: 260–286. 10.1111/1541-4337.13065. 36385735

[fsn370103-bib-0046] Romano, A. , L. D. Luca , and R. Romano . 2024. “Effects of Germination Time on the Structure, Functionality, Flavour Attributes, and In Vitro Digestibility of Green Altamura Lentils (*Lens culinaris* Medik.) Flour.” Food & Function 15, no. 7: 3539–3551. 10.1039/D3FO05758E. 38465882

[fsn370103-bib-0047] Samtiya, M. , R. E. Aluko , and T. Dhewa . 2020. “Plant Food Anti‐Nutritional Factors and Their Reduction Strategies: An Overview.” Food Production, Processing and Nutrition 2, no. 1: 6. 10.1186/s43014-020-0020-5.

[fsn370103-bib-0048] Setia, R. , Z. Dai , M. T. Nickerson , E. Sopiwnyk , L. Malcolmson , and Y. Ai . 2019. “Impacts of Short‐Term Germination on the Chemical Compositions, Technological Characteristics and Nutritional Quality of Yellow Pea and Faba Bean Flours.” Food Research International 122: 263–272. 10.1016/j.foodres.2019.04.021. 31229080

[fsn370103-bib-0049] Shi, J. , K. Arunasalam , D. Yeung , Y. Kakuda , G. Mittal , and Y. Jiang . 2004. “Saponins From Edible Legumes: Chemistry, Processing, and Health Benefits.” Journal of Medicinal Food 7, no. 1: 67–78. 10.1089/109662004322984734. 15117556

[fsn370103-bib-0050] Shi, L. , S. D. Arntfield , and M. Nickerson . 2018. “Changes in Levels of Phytic Acid, Lectins and Oxalates During Soaking and Cooking of Canadian Pulses.” Food Research International 107: 660–668. 10.1016/j.foodres.2018.02.056. 29580532

[fsn370103-bib-0051] Shi, L. , K. Mu , S. D. Arntfield , and M. T. Nickerson . 2017. “Changes in Levels of Enzyme Inhibitors During Soaking and Cooking for Pulses Available in Canada.” Journal of Food Science and Technology 54, no. 4: 1014–1022. 10.1007/s13197-017-2519-6. 28303052 PMC5336459

[fsn370103-bib-0052] Singh, B. , J. P. Singh , N. Singh , and A. Kaur . 2017. “Saponins in Pulses and Their Health Promoting Activities: A Review.” Food Chemistry 233: 540–549. 10.1016/j.foodchem.2017.04.161. 28530610

[fsn370103-bib-0053] Singleton, V. L. , and J. A. Rossi . 1965. “Colorimetry of Total Phenolics With Phosphomolybdic‐Phosphotungstic Acid Reagents.” American Journal of Enology and Viticulture 16, no. 3: 144–158.

[fsn370103-bib-0054] Sinkovič, L. , B. Pipan , F. Šibul , I. Nemeš , A. Tepić Horecki , and V. Meglič . 2022. “Nutrients, Phytic Acid and Bioactive Compounds in Marketable Pulses.” Plants 12, no. 1: 170. 10.3390/plants12010170. 36616298 PMC9824021

[fsn370103-bib-0055] Sofi, S. A. , S. Rafiq , J. Singh , et al. 2023. “Impact of Germination on Structural, Physicochemical, Techno‐Functional, and Digestion Properties of Desi Chickpea (*Cicer arietinum* L.) Flour.” Food Chemistry 405: 135011. 10.1016/j.foodchem.2022.135011. 36442241

[fsn370103-bib-0056] Stone, A. , K. Waelchli , B. Çabuk , et al. 2021. “The Levels of Bioactive Compounds Found in Raw and Cooked Canadian Pulses.” Food Science and Technology International 27, no. 6: 528–538. 10.1177/1082013220973804. 33222551

[fsn370103-bib-0057] Tajoddin, M. D. , M. Shinde , and J. Lalitha . 2011. “In Vivo Reduction the Phytic Acid Content of Mung Bean (*Phaseolus aureus* L) Cultivars During Germination.” American‐Eurasian Journal of Agricultural & Environmental Sciences 10, no. 1: 127–132.

[fsn370103-bib-0058] Urbano, G. , M. López‐Jurado , S. Frejnagel , et al. 2005. “Nutritional Assessment of Raw and Germinated Pea (*Pisum sativum* L.) Protein and Carbohydrate by In Vitro and In Vivo Techniques.” Nutrition 21, no. 2: 230–239. 10.1016/j.nut.2004.04.025. 15723753

[fsn370103-bib-0059] Xu, M. , Z. Jin , A. Peckrul , and B. Chen . 2018. “Pulse Seed Germination Improves Antioxidative Activity of Phenolic Compounds in Stripped Soybean Oil‐In‐Water Emulsions.” Food Chemistry 250: 140–147. 10.1016/j.foodchem.2018.01.049. 29412904

